# A novel knowledge-derived data potentizing method revealed unique liver cancer-associated genetic variants

**DOI:** 10.1186/s40246-019-0213-7

**Published:** 2019-07-04

**Authors:** Naznin Sultana, Mijanur Rahman, Sanat Myti, Jikrul Islam, Md. G. Mustafa, Kakon Nag

**Affiliations:** 1 Globe Biotech Limited, Plot No # 3/KA, Tejgaon Industrial Area, Dhaka, 1208 Bangladesh; 2 Bangabandhu Sheikh Mujib Medical University, Shahbagh, Dhaka, 1000 Bangladesh

**Keywords:** NGS, Exome sequencing, Cancer genomics, Personalized medicine, Novel variants

## Abstract

**Background:**

Next-generation sequencing (NGS) has been advancing the progress of detection of disease-associated genetic variants and genome-wide profiling of expressed sequences over the past decade. NGS enables the analyses of multiple regions of a genome in a single reaction format and has been shown to be a cost-effective and efficient tool for root-cause analysis of disease and optimization of treatment. NGS has been leading global efforts to device personalized and precision medicine (PM) in clinical practice. The effectiveness of NGS for the aforementioned applications has been proven unequivocal for multifactorial diseases like cancer. However, definitive prediction of cancer markers for all types of diseases and for global populations still remains highly rewarding because of the diversity of cancer types and genetic variants in human.

**Results:**

We performed exome sequencing of four samples in quest of critical genetic factor/s associated with liver cancer. By imposing knowledge-based filter chains, we have revealed a panel of genetic variants, which are unrecognized by current major genomics data repositories. Total 20 MNV-induced, 5 INDEL-induced, and 31 SNV-induced neoplasm-exclusive genes were revealed through NGS data acquisition followed by data curing with the application of quality filter chains. Liver-specific expression profile of the identified gene pool is directed to the selection of 17 genes which could be the as likely causative genetic factors for liver cancer. Further study on expression level and relevant functional significance enables us to identify and conclude the following four novel variants, viz., c.416T>C (p.Phe139Ser) in SORD, c.1048_1049delGCinsCG (p.Ala350Arg) in KRT6A, c.1159G>T (p.Gly387Cys) in SVEP1, and c.430G>C (p.Gly144Arg) in MRPL38 as a critical genetic factor for liver cancer.

**Conclusion:**

By applying a novel data prioritizing rationale, we explored a panel of previously unaddressed liver cancer-associated variants. These findings may have an opportunity for early prediction of neoplasm/cancer in liver and designing of relevant personalized/precision liver cancer therapeutics in clinical practice. Since NGS protocol is associated with tons of non-specific mutations due to the variation in background genetic makeup of subjects, therefore, our method of data curing could be applicable for more effective screening of global genetic variants related to disease onset, progression, and remission.

**Electronic supplementary material:**

The online version of this article (10.1186/s40246-019-0213-7) contains supplementary material, which is available to authorized users.

## Background

Cancer is a multifactorial disease mostly influenced by genetics and environmental factors. At the genetic level, a cancerous phenomenon results from the accumulation of genomic alterations leading to the dysregulation of cell proliferation, regeneration, and apoptosis [9]. Hepatocellular carcinoma (HCC) is the fifth most common human cancer among different types of cancer, with approximately 750,000 new cases occurring worldwide each year [6]. About 85% of hepatocellular carcinoma (HCC) patients are from developing countries, such as Southeast Asia and sub-Saharan Africa [25], and worldwide death for liver cancer is 50%. One clinical finding suggests that the older HCC patients more often face hepatomegaly, vascular spider, and pleural effusion [29]. The treatment strategies for patients with HCC include surgery, radiation, chemotherapy, liver transplantation, and targeted therapies. Although there have been a lot of improvements in the diagnosis and treatment protocols, the death rates are increasing for patients with HCC. The majority of studies showed that a 5-year survival rate is less than 5% [18]. As of to date, “PubMed” searches of the phrases “Cancer genomics” and “NGS sequencing” have revealed more than 8000 and 3100 hits, respectively. That means around 40% of total publications related to NGS are dealt with cancer. This is clearly suggesting the popularity and acceptability of NGS technology in the field of cancer genomics. This study has revealed enormous information regarding the cause-and-effect relationship of gene and cancer in all phases of the disease, e.g., cancer onset, cancer progression, and cancer remission. Particularly, cancer prediction using the genetic markers has become a boon for humanity as it propels the success rate in cancer treatment by early detection of the disease. Recent advance in NGS technique has introduced the notion like precision cancer medicine and precision cancer genomics by the start of this century [8]. Nowadays, NGS-based whole-exome sequencing enables the scientific community to look closely the detail of genetic aberration profile and associated dysregulated signaling pathways. NGS techniques ultimately revolutionize precision cancer medicine through anticancer drug development and targeted therapy [28].

Cancer-associated genomic alterations are more global than local in nature [13]. The gross chromosomal structure alterations by amplification, deletion, translocation, and/or inversion of chromosomal segments are considered as common characteristics of cancer genomes [12]. The heterogeneous nature of cancers at a spatial and temporal scale has diversified the cancerous genome at the individual level [3]. Significant numbers of studies with liver cancer background indicate that NGS plays a crucial role in cancer diagnosis, classification, and treatment [26]. Importantly, a comprehensive assessment of cancer genome-associated genetic alteration plays a critical role in predicting oncology drugs and therapeutic outcomes [5, 26]; these could have druggable as well as a novel target for drug discovery. Therefore, NGS is an important tool for both the clinician and scientific community.

NGS-based whole-exome sequencing of individuals as a tool of personal genomics is a recent trend in cancer research [27]. One of the crucial challenges associated with such practice is the analysis and extracting out the meaningful information from the overwhelming amount of data generated by NGS [24]. Lack of valid and precise data mining pipeline forces the scientist community to identify authentic variants during mutation analysis of cancer [17].

This study was aimed for identifying liver cancer-specific genetic variants using a knowledge-based filter chains associated with variant prioritizing protocols. Through these filter chains, a panel of previously unreported liver cancer-associated variants has been extracted from whole-exome sequencing (WES) data which has the potential to drive us for the development of novel therapeutics.

## Materials and methods

### Subject selection

NGS-based genomic landscape analysis was performed on a total of four human subjects from South Asian population: one metastatic cancer patient and three asymptomatic healthy subjects comprising two males and one female. The selected subjects were aged between 50 and 70 years old. Samples were collected following the institutional ethical policy. The clinicopathologic features of the neoplasm of a liver patient include hepatomegaly with a large space-occupying lesion (SOL) in the right lobe of the liver. Fine needle aspiration from SOL of the liver showed numerous malignant cells with finely granular chromatin pattern with hemorrhagic background under microscopy. Additionally, elevated alpha-fetoprotein level (207.0 IU/mL) was estimated in the blood.

### Genomic DNA isolation

The analysis was performed on the genomic DNA extracted from the blood samples of patients. An automated platform (MagMAX™ Express-96 Magnetic Particle Processor; Life Technologies, USA) was used to extract the genomic DNA from blood samples following the manufacturer’s instructions of MagMAX™ DNA Multi-Sample Kits (Life Technologies, USA). The quantity of the extracted DNA was estimated using Qubit™ dsDNA HS assay kit (Life Technologies, USA) in combination with Qubit™ fluorometer (Life Technologies, USA).

### Next-generation sequencing

Next-generation sequencing (NGS) was performed as previously described [4, 7] by Fujita and Damiati in 2017 and 2016, respectively. In brief, 100 ng of DNA was amplified for genomic library preparation using the exome enrichment kit (Ion AmpliSeq™, Life Technologies, USA) in order to sequence the key exonic regions (> 97% of CCDSs) of the genome. Ion Chef™ System (Life Technologies, USA) was used for template preparation and enrichment using Ion 540™ Kit – Chef (Life Technologies, USA). The same automated platform was used for loading Ion 540™ Chips with template-positive Ion Sphere™ Particles. Exome sequencing was performed on Ion S5™ XL Sequencer (Life Technologies, USA) with the loaded chips. Data analysis was done by Torrent Suite™ Software (v 5.2.2; Life Technologies, USA). Coverage analysis was performed using the Coverage Analysis plug-in (v5.2.0.9). Variant Caller plug-in (v5.2.0.34) was used for mutation/variant detection against the reference genome (hg19).

### Data filtering and prioritization

**Fig. 1:**
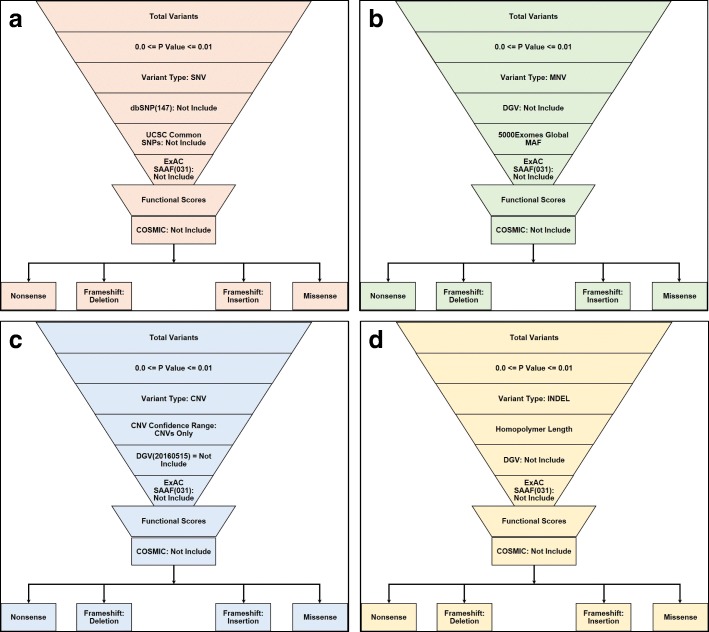
Filter chains applied for variant detection. Filter chains normalize common population mutations, known ethnic background, minor allele, and nonfunctional mutations. **a** SNP detection filter chain, **b** MNV detection filter chain, **c** CNV detection filter chain, and **d** INDEL detection filter chain

The Variant Call Format (VCF) and binary version of SAM (BAM) files for all samples were uploaded into Ion Reporter™ 5.10.2.0 (Life Technologies, USA) for data filtering and prioritization using variant-specific filter chain (Fig. 1) for identifying liver cancer-specific genetic variants. Total variants of each sample were detected by “Variant Caller” plug-in where a *p* value was 0.0–0.01. Despite using numbers of bioinformatics, data repositories retrieved variants through most extensive and curated servers and then categorized according to the variants type, and then imposing distinct variant-specific, customized filter chains. After that, “Exome Aggregation Consortium South Asian Allelic Frequency (ExAC SAAF)” hits were filtered out for the elimination of rare genetic variation for “South Asian” population.Remaining variants were then filtered by worse functional impact (SIFT score 0.0–0.05, PolyPhen score 0.85–1.0) and deleterious evolutionary distance (Grantham scores 101–215), respectively. Somatic mutations across the range of human cancers were excluded by applying “Catalog of Somatic Mutations in Cancer (COSMIC)” filter. After filtering all common variants, existing variants were classified according to the variant effect (e.g., nonsense, missense, frameshift insertion, and frameshift insertion mutations). “Single Nucleotide Polymorphism Database (dbSNP)” and “UCSC common SNPs” databases were applied for Single Nucleotide Variant (SNV) analysis; “Database of Genomic Variants (DGV)” and “5000Exomes” databases were applied for Multiple Nucleotide Variant (MNV) analysis; CNV confidence range and DGV databases were applied for Copy Number Variant (CNV) analysis; homopolymer length filter and DGV database were applied for Insertion or Deletion polymorphism (INDEL) analysis. Variants match with dbSNP, UCSC common SNPs, DGV and 5000Exomes database were excluded for downstream prioritization.

### Data selection for exclusive mutation

With the variant pools obtained from database analyses, data were curated for finding intra-subject match hits at least 100× coverage. A variant was considered for neoplasm-specific if and only if it occurred exclusively in GBNGS011 subject. The hits were then screened for liver-specific protein expression profile, and spatial functional and biological significance through comparison of “GeneCards” entries.

## Results

### Coverage analysis and variant detection

**Fig. 2:**
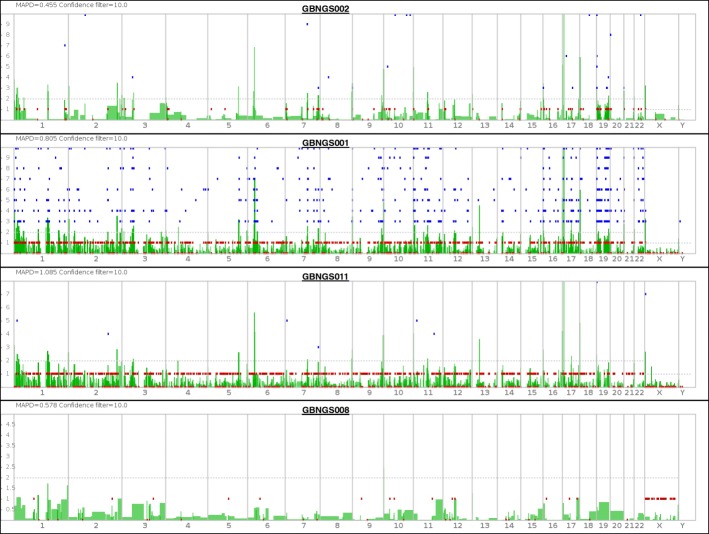
Whole-exome sequencing (WES) landscape constructed with Ion Reporter™ Genomic Viewer (IRGV). Here, “*x*”-axis indicate chromosome number and “*y*”-axis indicates confidence filter for CNV

The whole-exome sequencing (WES) data from four subjects were aligned against the reference genome hg19 for the analysis of coverage and detection of variants (Fig. 2) for probable incidental findings with a confidence level (Table 1).

**Table 1:** Analysis of coverage and variant detection

Sample (accession no.)	On target (%)	Mean depth	Variants
GBNGS001 (SRR8293457)	92.63	170.7	35,635
GBNGS002 (SRR8293456)	96.85	233.4	39,339
GBNGS008 (SRR8293455)	92.46	30.79	23,197
GBNGS011 (SRR8293454)	90.88	42.79	25,842

The range of the mean depth of coverage was 30–233. The sequence from GBNGS011 has the lowest percentage of mapped read. GBNGS011 is aligned on target with minimal variants (25842) calls, and GBNGS002 is aligned on target with maximum variant (39339) calls (see Table 1).

### SNV detection

**Fig. 3:**
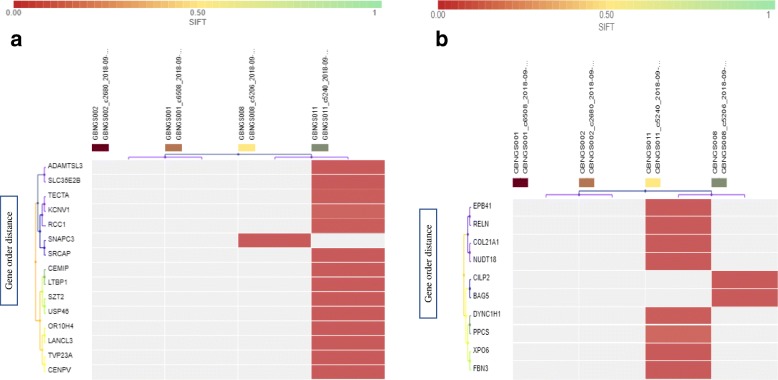
Heat map of SNP variant impacts. **a** SNP with frameshift Insertion (SIFT score 0.00–1). **b** SNP with frameshift deletion (SIFT score 0.00–1). Worse functional impact SIFT score ranges from 0.00 represents a deleterious effect in genes to 1 represents tolerated effect in genes. SIFT score ranges from 0.00 (deleterious) to 1.0 (tolerated). Variants with scores closer to 0.00 are more confidently predicted to be deleterious. Variants with scores 0.05 to 1 are predicted to be tolerated (benign). Variants with scores very close to 1 are more confidently predicted to be tolerated. Horizontal axis represents the gene order distance

The exome data from four subjects were filtered through SNP detection filter chain that consists of seven different filters (Fig. 1a). SNP detection filter chain filtered 411 SNV (Additional file 1: Tables S1, S2, and S3) from 121,556 variants associated with 400 genes. All the variants were recognized as missense mutation by default. Besides, frameshift deletion mutations were detected in 15 genes (*CDK11B*, *RCC1*, *SZT2*, *LTBP1*, *USP46*, *KCNV1*, *TECTA*, *CEMIP*, *ADAMTSL3*, *TVP23A*, *SRCAP*, *CENPV*, *OR10H4*, and *LANCL3*). GBNGS011 possesses almost all these mutated genes except *SNAPC3* that was found only in GBNGS008 (Fig. 3a). Among these, we retrieved 10 genes with SNV-associated frameshift insertion mutation, which includes *EPB41*, *PPCS*, *COL21A1*, *RELN*, *NUDT18*, *DYNC1H1*, *BAG5*, *XPO6*, *FBN3*, and *CILP2* (Fig. 3b). Among the study cohort, GBNGS008 carried a mutation on *CILP2* and *BAG5* genes and the rest of the mutations were carried only by GBNGS011. No nonsense mutation was detected by SNP detection filter chain.

### MNV detection

**Fig. 4:**
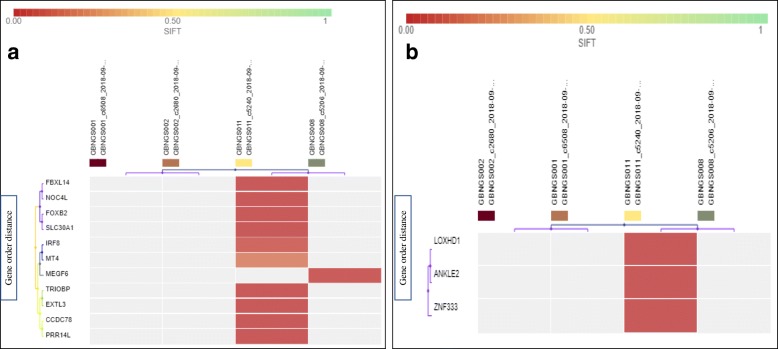
Heat map of MNV variant impacts. **a** MNV with frameshift insertion (SIFT score 0.00–1). **b** SNP with nonsense mutation (SIFT score 0.00–1). Worse functional impact SIFT score ranges from 0.00 represents a deleterious effect in genes to 1 represents tolerated effect in genes. Variants with scores closer to 0.00 are more confidently predicted to be deleterious. Variants with scores 0.05 to 1 are predicted to be tolerated (benign). Variants with scores very close to 1 are more confidently predicted to be tolerated. Horizontal axis represents gene order distance

MNV detection filter chain (Fig. 1b) generated 222 variants from 121,556 variants associated with 219 genes. At first, 222 variants were recognized as missense mutation (Additional file 1: Table S4). MNV-associated frameshift insertion mutation filtering analysis revealed that except *MEGF6* gene from GBNGS008, all other genes, viz., *SLC30A1*, *EXTL3*, *FOXB2*, *FBXL14*, *NOC4L*, *CCDC78*, *MT4*, *IRF8*, *PRR14L*, and *TRIOBP* were from GBNGS011 (Fig. 3a). We have also observed MNV-induced nonsense mutation in *ZNF333*, *ANKLE2*, and *LOXHD1* genes (Fig. 4b) in GBNGS011. A total of 42 genes were found to be associated with frameshift deletion mutation (Additional file 1: Table S5) due to MNV. Among these gene pools, GBNGS008, GBNGS001, and GBNGS002 carried a mutation in *KLHL5* genes, *PIF1* gene, and *DDX60* gene, respectively. The rest of the mutations (on *ATR*, *DNAH10*, *ARID4B*, *ADAMTS7*, *JMJD6*, *MAP3K6*, *KLK12*, *SF3A3*, *B4GALT3*, *HSD17B3*, *SCG5*, *PFAS*, *ARSD*, *NOS2*, *KCND2*, *CUBN*, *MUC2*, *WDCP*, *AHNAK2*, *SLC25A29*, *DNAH2*, *TJP3*, *MEPCE*, *PKD2*, *TXNDC11*, *GTF3A*, *MYO15A*, *PHF1*, *RBM4*, *RBM14-RBM4*, *ATP5J2-PTCD1*, *PTCD1*, *IFT46*, *NRCAM*, *CHPF2*, *SH2B1*, *METTL23*, *SNN*, and *MTIF3* genes) were in GBNGS011.

### CNV detection

CNV detection filter chain (Fig. 1c) primarily filtered 85 CNV from 121,556 variants and then applying a COSMIC filter which ultimately nullified the CNV output.

### INDEL detection

**Fig. 5:**
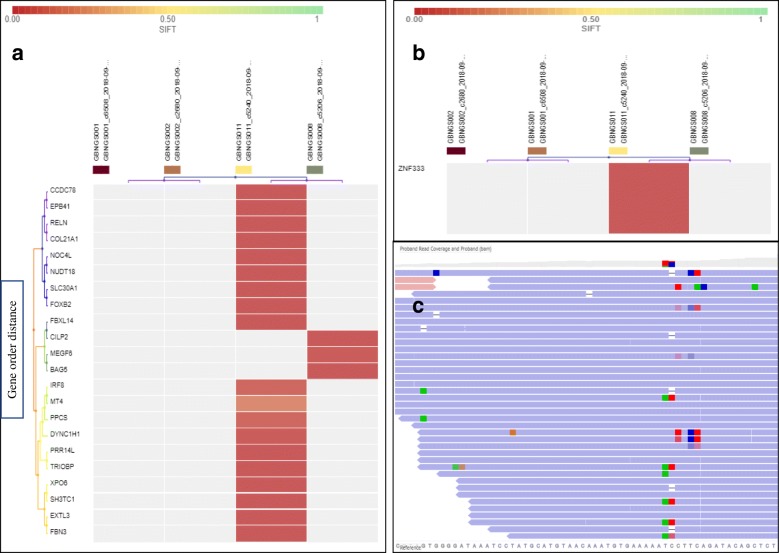
Heat map of INDEL variant impacts. **a** INDEL with frameshift insertion (SIFT score 0.00–1). **b** INDEL with nonsense mutation (SIFT score 0.00–1). Worse functional impact SIFT score ranges from 0.00 represents a deleterious effect in genes to 1 represents tolerated effect in genes. Variants with scores closer to 0.00 are more confidently predicted to be deleterious. Variants with scores 0.05 to 1 are predicted to be tolerated (benign). Variants with scores very close to 1 are more confidently predicted to be tolerated. Horizontal axis represents the gene order distance. **c** IRGV visualization for locus chr19:14829263 of GBNGS011

INDEL detection filter chain (Fig. 1d) resulting in 95 variants out of 121,556 associated with 95 involved genes. At first, 95 variants were recognized as missense mutation (Additional file 1: Table S4). INDEL-induced frameshift insertion mutation affected 22 genes present in GBNGS008 and GBNGS011. Among reported INDEL-associated frameshift insertion mutation-inflicted genes, three genes (*MEGF6*, *EPB41*, *PPCS*) were found in GBNGS008 and the rest 17 genes (*SLC30A1*, *SH3TC1*, *COL21A1*, *RELN*, *NUDT18*, *EXTL3*, *FOXB2*, *FBXL14*, *NOC4L*, *DYNC1H1*, *BAG5*, *CCDC78*, *XPO6*, *MT4*, *IRF8*, *FBN3*, *CILP2)* were found in GBNGS011. There was only one GBNGS011-exclusive INDEL-induced nonsense mutation in *ZNF333* gene (Fig. 5b). A total of 42 genes were detected with INDEL-associated frameshift deletion mutations (Additional file 1: Table S5). All INDEL-incurring frameshift deletion mutations were found in GBNGS011, except *DDX60* in GBNGS001, *PIF1* in GBNGS002, and *KLHL5* in GBNGS008.

### Neoplasm-exclusive mutations

**Fig. 6:**
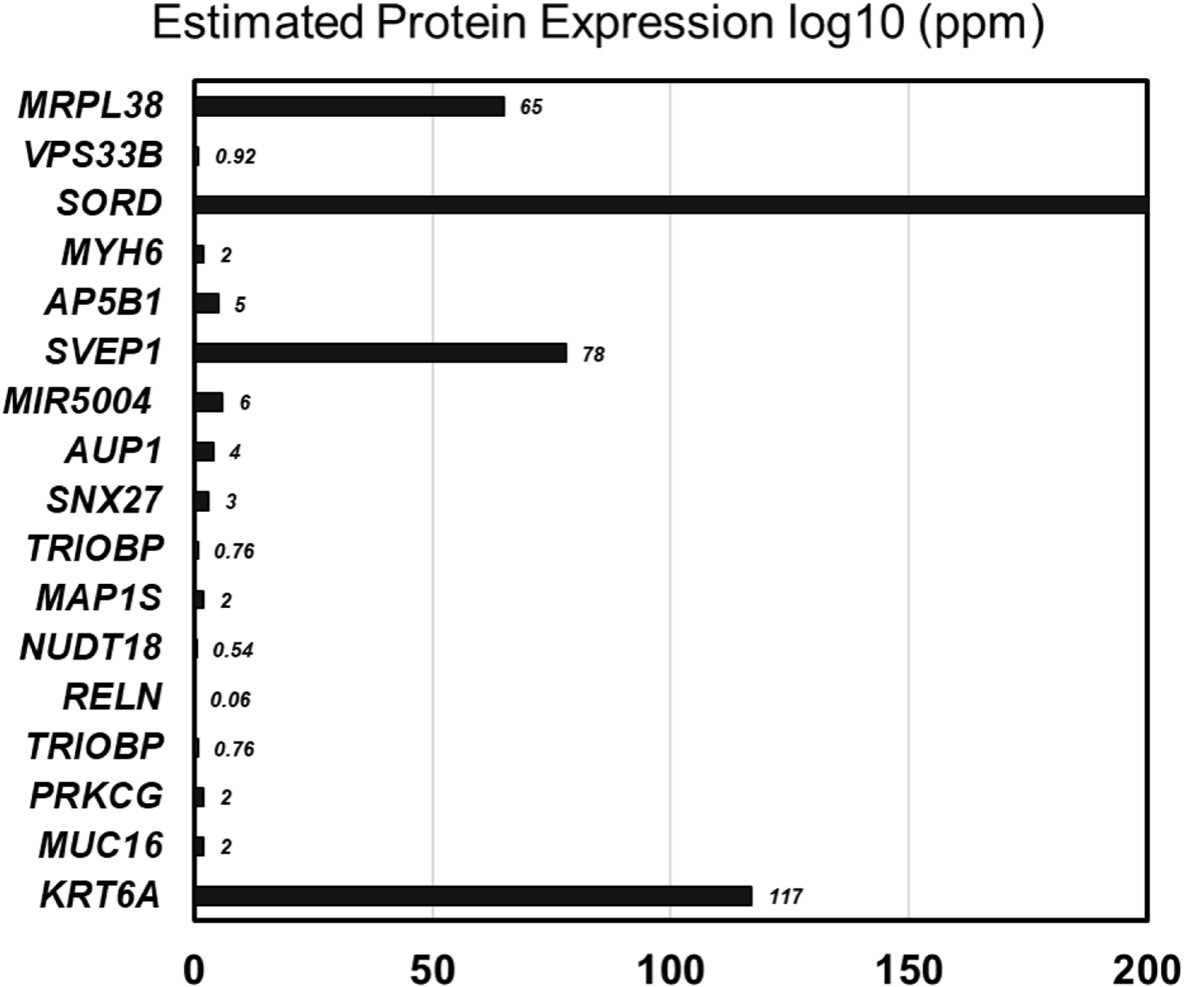
Tissue-specific expression profile of 17 neoplasm-exclusive genes in the liver. The data was congregated from “GeneCard”; *x*-axis indicates the level of protein expression and *y*-axis indicates the gene name

The combination of results from different filter chains revealed neoplasm-exclusive SNV-induced mutation in 31 genes, MNV-induced mutation in 20 genes, and INDEL-induced mutation in five genes (Additional file 1: Table S1), respectively. Among these candidates, as per “GeneCards” entry 17 genes, viz., *KRT6A*, *MUC16*, *PRKCG*, *TRIOBP*, *RELN*, *NUDT18*, *MAP1S*, *SNX27*, *AUP1*, *MIR5004*, *SVEP1*, *SORD*, *VPS33B*, *MRPL38*, *AP5B1*, and *MYH6* showed liver-specific expression (Fig. 6).

## Discussion

In this study, we performed NGS-based exome sequencing of a liver neoplasm patient against age-matched three asymptomatic subjects where hg19 was used as a reference genome for alignment. This experiment resulted in a total of 121,556 variant calls. We figure out a panel of variants for liver cancer through customized filter chain over 121,556 variants. These variants were so far unrecognized by major genomics data repositories.

This study cohort includes one liver neoplasm patient as a primary target. The objective of this study was figuring out the extent of personalized cancer associate variant profile. A group of three subjects was selected as a negative control for eliminating the regional population-associated variants. We applied knowledge-based stringent filter chains for overcoming the shortcomings of the small negative control group (*n* = 3). Tumor/cancer-associated somatic variant profile has prognostic value over tumor regression besides its rudimentary diagnostic use [14]. It is not surprising that the full repertoire of cancer-associated variants is still evolving [21]. Interestingly, quite a good portion of this diversity resulted from the ethnic and geographic background [23]. Thus, a comparison of normal to neoplastic DNA sequence theoretically allows more accurate identification of somatic changes [8].

A seven-stage filter chain was applied on the entire variant pool. The ultimate target of setting such filtering algorithm was to ensure knowledge-driven variant prioritization exclusive to neoplasm. Two distinct principles were considered for setting the entire filter layer: (1) elimination of population-based common variants and (2) inclusion of functionally significant and unreported cancer variants. Irrespective to the objective, a variant call above 99% confidence was subjected to the subsequent data-prioritization workflow.

The dbSNP, UCSC common SNPs, DGV, and 5000Exomes database were allocated within filter chains for achieving the first goal. The dbSNP and UCSC common SNP annotation expunged neutral and known phenotypes corresponding polymorphisms from the variant pool. The DGV hits identified structural variation in the human genome present in healthy samples whereas 5000Exomes Global MAF is the database of global minor allele frequencies. The cancer risk and treatment outcomes often show the population-based variation that largely attributed to genetic and environmental variation [11]. We have applied “ExAC SAAF” as population-based variant removal filter to overcome such effect. Indeed, the evolutionary forces govern the mutational frequencies across populations to shape the genetic diversity and ultimately contribute to ethnic and geographic differences [24]. Therefore, sorting out genetic diversity common to the global population as well as a particular ethnic group was included in the filter chain as exclusive variant prioritization strategies.

After exclusion of possible variants, we took functional relevance as a second dimension tool for identification of non-relevant variant exclusion. The variants were selected through SIFT, PolyPhen, and Grantham score cutoff, which have been considered associated with worse functional impact on a protein and also damage evolutionary distance. Specific filter chains were applied thereafter for gathering COSMIC unmatched variant to call cancer exclusion variants. A typical WES-data generates large numbers of genetic variants [16]. Prioritization of the variants in the context of disease study incorporates the urge of sorting functional relevant variants [1]. Thus, fixing these two filets in the filter chain enabled searching disease-relevant variants.

A pool of 17 genes was selected from liver-specific expression profile. Identified genes are quite diversified in their biological significance and disease association [20]. *KRT6A* encodes for keratin 6A and involved in wound healing; defects in this gene primarily leads to hypertrophic nail dystrophy (pachyonychia congenita 3 and pachyonychia congenita 1). Cell surface-associated Mucin 16 (*MUC16*) is used as a marker for different cancers and associated with an ovarian cyst. Protein Kinase C Gamma (*PRKCG*) is a member of serine- and threonine-specific protein kinase family that phosphorylates p53/TP53 and promotes p53/TP53-dependent apoptosis in response to DNA damage. *TRIOBP* encodes for TRIO and F-actin-binding protein. By interacting with the trio, *TRIOBP* controls actin cytoskeleton organization, cell motility, and cell growth. Reelin, encoded by *RELN*, regulates cell-cell interactions and modulates cell adhesion. Nudix hydrolase 18 (*NUDT*) is linked to purine metabolism. Microtubule-associated protein (MAP1S) mediates mitochondrial aggregation and consequential apoptosis. Sorting Nexin Family Member 27 (SNX27) is involved in recycling of internalized transmembrane proteins. *AUP1* encodes for lipid droplet regulating VLDL assembly factor, a protein that plays an essential role in the quality control of misfolded proteins in the endoplasmic reticulum and lipid droplet accumulation. *MIR5004* is an RNA gene that codes for MicroRNA 5004 which belongs to the miRNA class. This miRNA is affiliated with RET proto-oncogene signaling. *SVEP1* encodes “EGF and Pentraxin Domain Containing 1”. SVEP1 is associated with *calcium ion binding* and *chromatin binding*. Diseases related with sorbitol dehydrogenase (SORD) include cataract and microvascular complications of diabetes 5. *MRPL38* encodes for Mitochondrial Ribosomal Protein L38 and related to organelle biogenesis and maintenance and mitochondrial translation. The protein encoded by *AP5B1* (adaptor-related protein complex 5 subunit beta 1) is involved with hereditary spastic paraplegia. Myosin heavy chain 6 (MYH6) is associated with ERK signaling and cytoskeleton remodeling. Defect in myosin heavy chain 6 causes atrial septal defect 3 and cardiomyopathy.

Among these 17 genes, four genes showed the highest level of expression: *MRPL38*, *SORD*, *SVEP1*, and *KRT6A*. Though direct experimental data was unavailable about the association of these genes with liver neoplasm, data mining showed indirect association of the target gene pools with cancerous events. For example, mitochondrial ribosomal protein L38 (MRPL38) has been reported to be overexpressed (~ 4 times) in precursor T cell lymphoblastic leukemia (pre-T LBL) [10]. SORD expression and activity was upregulation in colorectal adenomas whereas SORD knockdown significantly blocked epithelial-to-mesenchymal transition (EMT) [17, 22]. The cell adhesion molecule SVEP1 can induce EMT and associated with disseminating cancer cells to secondary organ [2]. An upregulation of *KRT6A* gene product has been reported in non-melanoma skin cancer [15]. It is noteworthy to mention that the cancer patient was graded to be metastatic whereas two of the candidate genes (*SORD* and *SVEP1*) were able to induce EMT.

A number of WES data analysis workflow is available to help extract useful information about disease-associated variants. Some of the pipelines emphasizes the variant annotation according to the variant effect that is conserved to our present proposed workflow. Unlike most of the data analysis platform, a number of public databases were incorporated in the proposed filter chain to figure out the unreported variants. The workflow designed and executed in this study enabled us to detect a panel of variants unreported in cancer database, which needs a functional study for further characterization.

Whole-exome NGS enables adopting effective and safer therapeutic decision targeting specific genetic alterations [19]. As a matter of fact, the data analysis pipeline used in this study was not free from the generic drawback for WES data analysis. The data analysis used a number of public databases whereas public databases containing variant information are not complete and error-free [1]. Though changing the filter sequence does not influence the ultimate filtering outcome, the sequence of the filter in the filter series is not warranted. Additionally, the filter chain possesses variant-specific database involvement that can be only explained by scientific justification with experimental proof.

“Inter-individual variability and tumor heterogeneity” is a well-known phenomenon in cancer genomics research [5]. Scientists have been focusing on “sera biomarkers” or “cancer hotspot-dependent” diagnostics for cancer detection. These types of diagnostic techniques are suitable for later/developed stage of disease onset, and efficacy of existing treatments for cancer are not very efficient. Therefore, a predictive diagnostics methodology would be very rewarding for the prognosis of cancer in a subject before the onset of the disease. Unlike cancerous tissue sample (either solid tumor or metastatic tissue) where genetic variability is observed [5], we relied on germline DNA for identification of causative markers for liver cancer.

Our subjects were identified and confirmed using available medical diagnostic protocols. We used these subjects with the confirmatory status of either diseased or healthy condition, collected their germline DNA from peripheral blood mononuclear cell (PBMC), and used a reversed engineering approach to reveal causative relevant functional mutations for this disease. The result of the method will provide an opportunity for foretelling the emergence of the disease at later time of life of a subject, so that a time-dependent confirmatory test program can be assigned. This approach will ensure the earliest confirmation of cancer onset and will provide the best efficacy for the treatment. If appropriate, lifestyle planning regimen can be adopted to avert or delay disease onset.

In summary, this research reiterates/votes for the importance of personalized cancer genomics as a tool for precision cancer management. The most important outcome of this study is a panel of previously unaddressed cancer-associated variants applying a novel data prioritizing rationale. The scientific community working with NGS platform can take the opportunity from this advantageous data prioritizing strategy for searching subject-exclusive mutation.

## Additional file

Additional file 1: **Table S1.** SNV-induced neoplasm-exclusive mutations. **Table S2.** INDEL-induced neoplasm-exclusive mutations. **Table S3.** SNP-induced neoplasm-exclusive mutations. **Table S4.** SIFT score of total SNP sorted by SNP detection filter chain. **Table S5.** SIFT score of total MNV sorted by MNV detection filter chain. **Table S6.** Splice variant impact of 42 genes incurred with frameshift deletion mutation due to MNV. **Table S7.** SIFT score of total INDEL sorted by INDEL detection filter chain. (DOCX 110 kb)

## Data Availability

WGS BAM files from this study have been submitted to SRA (http://www.ncbi.nlm.nih.gov/sra) under accession number SRR8293454, SRR8293455, SRR8293456, and SRR8293457.

## Abbreviations

BAM: Binary version of SAM
CNV: Copy number variant
COSMIC: Catalog of Somatic Mutations in Cancer
dbSNP: Single Nucleotide Polymorphism Database
DGV: Database of Genomic Variants
ExAC SAAF: Exome Aggregation Consortium South Asian Allelic Frequency
INDEL: Insertion or deletion polymorphism
IRGV: Ion Reporter™ Genomic Viewer
MNV: Multiple-nucleotide variant
NGS: Next-generation sequencing
PG: Pharmacogenomics
PM: Personalized medicine
SNV: Single nucleotide variant
SOL: Space-occupying lesions
VCF: Variant Call Format
VLDL: Very low-density lipoprotein
WES: Whole-exome sequencing
