# Supplementary material for: A novel knowledge-derived data potentizing method revealed unique liver cancer-associated genetic variants
Source: Hum Genomics. 2019 Jul 4;13:30. doi: 10.1186/s40246-019-0213-7 (PMC6610914; doi:10.1186/s40246-019-0213-7)
Supplement: Supplementary file 1 — Table S1. SNV-induced neoplasm-exclusive mutations. Table S2. INDEL-induced neoplasm-exclusive mutations. Table S3. SNP-induced neoplasm-exclusive mutations. Table S4. SIFT score of total SNP sorted by SNP detection filter chain. Table S5. SIFT score of total MNV sorted by MNV detection filter chain. Table S6. Splice variant impact of 42 genes incurred with frameshift deletion mutation due to MNV. Table S7. SIFT score of total INDEL sorted by INDEL detection filter chain. (DOCX 110 kb) [file 40246_2019_213_MOESM1_ESM.docx]

**A novel knowledge-derived data potentizing approach for extracting unreported cancer associated variants**

**Naznin Sultana^1,*^, Mohammad M. Rahman^1^, Sanat Myti^1^, Md. J. Islam^1^, Md. G. Mustafa^2^, Kakon Nag^1,*^**

^1^ Globe Biotech Limited, Plot No # 3/KA, Tejgaon Industrial Area, Dhaka-1208, Bangladesh.

^2^ Bangabandhu Sheikh Mujib Medical University, Shahbagh, Dhaka-1000, Bangladesh.

* To whom correspondence should be addressed

E-mail address: [naznin004@globe-biotech.com](mailto:Kakonpoly@gmail.com), kakonpoly@yahoo.com

Telephone no: +8801709631831

**Additional file table S1: SNV-induced neoplasm exclusive mutations**

| [**Locus**](https://ionreporter.thermofisher.com/ir/secure/analyses/visualization.html) | **Ref** | **Type** | **Variant Frequency** | **Genes** | **GBNGS002** | **GBNGS001** | **GBNGS011** | **GBNGS008** | **Amino Acid Change** | **Coding** |
| --- | --- | --- | --- | --- | --- | --- | --- | --- | --- | --- |
| [chr1:16374492](https://ionreporter.thermofisher.com/ir/secure/analyses/visualization.html) | CT | MNV | 0.25 | [CLCNKB](http://www.genenames.org/cgi-bin/gene_symbol_report?hgnc_id=2027) |  |  | CT/TG |  | p.Leu151Cys | c.451_452delCTinsTG |
| [chr1:16456056](https://ionreporter.thermofisher.com/ir/secure/analyses/visualization.html) | CG | MNV | 0.25 | [EPHA2](http://www.genenames.org/cgi-bin/gene_symbol_report?hgnc_id=3386) |  |  | CG/GC |  | p.Ser899_Gly900delinsArgArg | c.2697_2698delCGinsGC |
| [chr1:28285186](https://ionreporter.thermofisher.com/ir/secure/analyses/visualization.html) | CTG | MNV | 0.25 | [SMPDL3B ...(2)](javascript:void(0)) |  |  | CTG/GCT |  | p.Ala402_Gly403delinsGlyTrp, p.? | c.1205_1207delCTGinsGCT, c.-1395CTG>GCT |
| [chr1:117556034](https://ionreporter.thermofisher.com/ir/secure/analyses/visualization.html) | AT | MNV | 0.25 | [CD101](http://www.genenames.org/cgi-bin/gene_symbol_report?hgnc_id=5949) |  |  | AT/TA |  | p.Asp283Val | c.848_849delATinsTA |
| [chr1:154744620](https://ionreporter.thermofisher.com/ir/secure/analyses/visualization.html) | TG | MNV | 0.25 | [KCNN3](http://www.genenames.org/cgi-bin/gene_symbol_report?hgnc_id=6292) |  |  | TG/GT |  | p.Ser426_Lys427delinsArgGln | c.1278_1279delCAinsAC |
| [chr2:98828420](https://ionreporter.thermofisher.com/ir/secure/analyses/visualization.html) | GC | MNV | 0.25 | [VWA3B](http://www.genenames.org/cgi-bin/gene_symbol_report?hgnc_id=28385) |  |  | GC/CG |  | p.Ala589Arg | c.1765_1766delGCinsCG |
| [chr8:38369936](https://ionreporter.thermofisher.com/ir/secure/analyses/visualization.html) | GC | MNV | 0.25 | [C8orf86](http://www.genenames.org/cgi-bin/gene_symbol_report?hgnc_id=33774) |  |  | CG/CG |  | p.Ala214Arg | c.640_641delGCinsCG |
| [chr12:52884381](https://ionreporter.thermofisher.com/ir/secure/analyses/visualization.html) | GC | MNV | 0.25 | [KRT6A](http://www.genenames.org/cgi-bin/gene_symbol_report?hgnc_id=6443) |  |  | GC/CG |  | p.Ala350Arg | c.1048_1049delGCinsCG |
| [chr12:111760284](https://ionreporter.thermofisher.com/ir/secure/analyses/visualization.html) | CA | MNV | 0.25 | [CUX2](http://www.genenames.org/cgi-bin/gene_symbol_report?hgnc_id=19347) |  |  | CA/AC |  | p.Ser942_Lys943delinsArgGln | c.2826_2827delCAinsAC |
| [chr12:132271036](https://ionreporter.thermofisher.com/ir/secure/analyses/visualization.html) | GC | MNV | 0.25 | [SFSWAP](http://www.genenames.org/cgi-bin/gene_symbol_report?hgnc_id=10790) |  |  | GC/CG |  | p.Ala865Arg | c.2593_2594delGCinsCG |
| [chr16:776046](https://ionreporter.thermofisher.com/ir/secure/analyses/visualization.html) | CA | MNV,INDEL | 0.25 | [CCDC78](http://www.genenames.org/cgi-bin/gene_symbol_report?hgnc_id=14153) |  |  | AC/CAC |  | p.Gly34Trp, p.Gly34fs | c.99_100delTGinsGT, c.98_99insG |
| [chr16:2140532](https://ionreporter.thermofisher.com/ir/secure/analyses/visualization.html) | GC | MNV | 0.25 | [LOC105371049 ...(3)](javascript:void(0)) |  |  | GC/CG |  | p.Cys4066Ser | c.12197_12198delGCinsCG |
| [chr16:50338392](https://ionreporter.thermofisher.com/ir/secure/analyses/visualization.html) | TTG | MNV | 0.25 | [ADCY7](http://www.genenames.org/cgi-bin/gene_symbol_report?hgnc_id=238) |  |  | TTG/GCT |  | p.Phe497_Ala498delinsCysSer | c.1490_1492delTTGinsGCT |
| [chr16:75646243](https://ionreporter.thermofisher.com/ir/secure/analyses/visualization.html) | GC | MNV | 0.25 | [ADAT1](http://www.genenames.org/cgi-bin/gene_symbol_report?hgnc_id=228) |  |  | GC/CG |  | p.Ala314Arg | c.940_941delGCinsCG |
| [chr19:2102213](https://ionreporter.thermofisher.com/ir/secure/analyses/visualization.html) | TG | MNV | 0.25 | [AP3D1](http://www.genenames.org/cgi-bin/gene_symbol_report?hgnc_id=568) |  |  | TG/GT |  | p.Ser1202_Asn1203delinsArgHis | c.3606_3607delCAinsAC |
| [chr19:9057504](https://ionreporter.thermofisher.com/ir/secure/analyses/visualization.html) | GC | MNV | 0.25 | [MUC16](http://www.genenames.org/cgi-bin/gene_symbol_report?hgnc_id=15582) |  |  | GC/CG |  | p.Ala9981Arg | c.29941_29942delGCinsCG |
| [chr19:40440520](https://ionreporter.thermofisher.com/ir/secure/analyses/visualization.html) | AC | MNV | 0.25 | [FCGBP](http://www.genenames.org/cgi-bin/gene_symbol_report?hgnc_id=13572) |  |  | AC/GA |  | p.Gly2Val | c.5_6delGTinsTC |
| [chr19:47980177](https://ionreporter.thermofisher.com/ir/secure/analyses/visualization.html) | AC | MNV | 0.25 | [KPTN](http://www.genenames.org/cgi-bin/gene_symbol_report?hgnc_id=6404) |  |  | AC/CA |  | p.Gly294Val | c.881_882delGTinsTG |
| [chr19:54410064](https://ionreporter.thermofisher.com/ir/secure/analyses/visualization.html) | TC | MNV | 0.25 | [PRKCG](http://www.genenames.org/cgi-bin/gene_symbol_report?hgnc_id=9402) |  |  | CT/CT |  | p.Phe670Ser | c.2009_2010delTCinsCT |
| [chr22:38161790](https://ionreporter.thermofisher.com/ir/secure/analyses/visualization.html) | GC | MNV,INDEL | 0.25 | [TRIOBP](http://www.genenames.org/cgi-bin/gene_symbol_report?hgnc_id=17009) |  |  | CG/GCG |  | p.Trp2146_Leu2147delinsCysVal, p.Leu2147fs | c.6438_6439delGCinsCG, c.6439_6440i |

**Additional file table S2: INDEL-induced neoplasm exclusive mutations**

| [**Locus**](https://ionreporter.thermofisher.com/ir/secure/analyses/visualization.html?) | **Ref** | **Type** | **Variant Frequency** | **Genes** | **GBNGS002** | **GBNGS001** | **GBNGS011** | **GBNGS008** | **Amino Acid Change** | **Coding** |
| --- | --- | --- | --- | --- | --- | --- | --- | --- | --- | --- |
| [chr7:103276776](https://ionreporter.thermofisher.com/ir/secure/analyses/visualization.html?) | C | SNV,INDEL | 0.25 | [RELN](http://www.genenames.org/cgi-bin/gene_symbol_report?hgnc_id=9957) |  |  | A/CA |  | p.Gly737Cys, p.Gly737fs | c.2209G>T, c.2208_2209insT |
| [chr8:21965819](https://ionreporter.thermofisher.com/ir/secure/analyses/visualization.html?) | G | SNV,INDEL | 0.25 | [NUDT18](http://www.genenames.org/cgi-bin/gene_symbol_report?hgnc_id=26194) |  |  | C/GC |  | p.Cys67Trp, p.Cys67fs | c.201C>G, c.200_201insG |
| [chr16:776046](https://ionreporter.thermofisher.com/ir/secure/analyses/visualization.html?) | CA | MNV,INDEL | 0.25 | [CCDC78](http://www.genenames.org/cgi-bin/gene_symbol_report?hgnc_id=14153) |  |  | AC/CAC |  | p.Gly34Trp, p.Gly34fs | c.99_100delTGinsGT, c.98_99insG |
| [chr19:17836943](https://ionreporter.thermofisher.com/ir/secure/analyses/visualization.html?) | CG | INDEL,SNV | 0.25 | [MAP1S](http://www.genenames.org/cgi-bin/gene_symbol_report?hgnc_id=15715) |  |  | C/CT |  | p.Gly251Trp, p.Asp252fs | c.751G>T, c.754delG |
| [chr22:38161790](https://ionreporter.thermofisher.com/ir/secure/analyses/visualization.html?) | GC | MNV,INDEL | 0.25 | [TRIOBP](http://www.genenames.org/cgi-bin/gene_symbol_report?hgnc_id=17009) |  |  | CG/GCG |  | p.Trp2146_Leu2147delinsCysVal, p.Leu2147fs | c.6438_6439delGCinsCG, c.6439_6440i |

**Additional file table S3: SNP-induced neoplasm exclusive mutations**

| [**Locus**](https://ionreporter.thermofisher.com/ir/secure/analyses/visualization.html?) | **Ref** | **Type** | **Variant Frequency** | **Genes** | **GBNGS002** | **GBNGS001** | **GBNGS011** | **GBNGS008** | **Amino Acid Change** | **Coding** |
| --- | --- | --- | --- | --- | --- | --- | --- | --- | --- | --- |
| [chr1:36060012](https://ionreporter.thermofisher.com/ir/secure/analyses/visualization.html?) | T | SNV | 0.25 | [TFAP2E](http://www.genenames.org/cgi-bin/gene_symbol_report?hgnc_id=30774) |  |  | T/G |  | p.Phe355Cys | c.1064T>G |
| [chr1:41514530](https://ionreporter.thermofisher.com/ir/secure/analyses/visualization.html?) | C | SNV | 0.25 | [SCMH1](http://www.genenames.org/cgi-bin/gene_symbol_report?hgnc_id=19003) |  |  | C/G |  | p.Gly380Arg | c.1138G>C |
| [chr1:45270140](https://ionreporter.thermofisher.com/ir/secure/analyses/visualization.html?) | A | SNV | 0.25 | [PLK3](http://www.genenames.org/cgi-bin/gene_symbol_report?hgnc_id=2154) |  |  | A/T |  | p.Asp491Val | c.1472A>T |
| [chr1:151630799](https://ionreporter.thermofisher.com/ir/secure/analyses/visualization.html?) | T | SNV | 0.25 | [SNX27](http://www.genenames.org/cgi-bin/gene_symbol_report?hgnc_id=20073) |  |  | T/G |  | p.Phe211Cys | c.632T>G |
| [chr1:186646863](https://ionreporter.thermofisher.com/ir/secure/analyses/visualization.html?) | A | SNV | 0.25 | [PTGS2](http://www.genenames.org/cgi-bin/gene_symbol_report?hgnc_id=9605) |  |  | A/G |  | p.Phe186Ser | c.557T>C |
| [chr2:74044061](https://ionreporter.thermofisher.com/ir/secure/analyses/visualization.html?) | T | SNV | 0.25 | [C2orf78](http://www.genenames.org/cgi-bin/gene_symbol_report?hgnc_id=34349) |  |  | T/G |  | p.Phe904Cys | c.2711T>G |
| [chr2:74755429](https://ionreporter.thermofisher.com/ir/secure/analyses/visualization.html?) | C | SNV | 0.25 | [AUP1](http://www.genenames.org/cgi-bin/gene_symbol_report?hgnc_id=891) |  |  | C/G |  | p.Trp206Ser | c.617G>C |
| [chr5:147029950](https://ionreporter.thermofisher.com/ir/secure/analyses/visualization.html?) | C | SNV | 0.25 | [JAKMIP2 ...(2)](javascript:void(0)) |  |  | C/G |  | p.Arg263Pro | c.788G>C |
| [chr5:151235906](https://ionreporter.thermofisher.com/ir/secure/analyses/visualization.html?) | T | SNV | 0.25 | [GLRA1](http://www.genenames.org/cgi-bin/gene_symbol_report?hgnc_id=4326) |  |  | T/A |  | p.Asn172Ile | c.515A>T |
| [chr6:33405623](https://ionreporter.thermofisher.com/ir/secure/analyses/visualization.html?) | T | SNV | 0.25 | [MIR5004 ...(2)](javascript:void(0)) |  |  | T/G |  | p.Phe314Cys | c.941T>G |
| [chr7:103276776](https://ionreporter.thermofisher.com/ir/secure/analyses/visualization.html?) | C | SNV,INDEL | 0.25 | [RELN](http://www.genenames.org/cgi-bin/gene_symbol_report?hgnc_id=9957) |  |  | A/CA |  | p.Gly737Cys, p.Gly737fs | c.2209G>T, c.2208_2209insT |
| [chr7:128415810](https://ionreporter.thermofisher.com/ir/secure/analyses/visualization.html?) | A | SNV | 0.25 | [OPN1SW](http://www.genenames.org/cgi-bin/gene_symbol_report?hgnc_id=1012) |  |  | A/G |  | p.Phe12Ser | c.35T>C |
| [chr7:149521504](https://ionreporter.thermofisher.com/ir/secure/analyses/visualization.html?) | G | SNV | 0.25 | [SSPO](http://www.genenames.org/cgi-bin/gene_symbol_report?hgnc_id=21998) |  |  | G/C |  | p.Trp4528Ser | c.13583G>C |
| [chr8:21965819](https://ionreporter.thermofisher.com/ir/secure/analyses/visualization.html?) | G | SNV,INDEL | 0.25 | [NUDT18](http://www.genenames.org/cgi-bin/gene_symbol_report?hgnc_id=26194) |  |  | C/GC |  | p.Cys67Trp, p.Cys67fs | c.201C>G, c.200_201insG |
| [chr9:34256243](https://ionreporter.thermofisher.com/ir/secure/analyses/visualization.html?) | T | SNV | 0.25 | [KIF24](http://www.genenames.org/cgi-bin/gene_symbol_report?hgnc_id=19916) |  |  | T/A |  | p.Asn1121Ile | c.3362A>T |
| [chr9:113275350](https://ionreporter.thermofisher.com/ir/secure/analyses/visualization.html?) | C | SNV | 0.25 | [SVEP1](http://www.genenames.org/cgi-bin/gene_symbol_report?hgnc_id=15985) |  |  | C/A |  | p.Gly387Cys | c.1159G>T |
| [chr11:6648608](https://ionreporter.thermofisher.com/ir/secure/analyses/visualization.html?) | C | SNV | 0.25 | [DCHS1](http://www.genenames.org/cgi-bin/gene_symbol_report?hgnc_id=13681) |  |  | C/G |  | p.Gly1888Arg | c.5662G>C |
| [chr11:65546579](https://ionreporter.thermofisher.com/ir/secure/analyses/visualization.html?) | G | SNV | 0.25 | [AP5B1](http://www.genenames.org/cgi-bin/gene_symbol_report?hgnc_id=25104) |  |  | G/T |  | p.Ala462Asp | c.1385C>A |
| [chr11:68748085](https://ionreporter.thermofisher.com/ir/secure/analyses/visualization.html?) | C | SNV | 0.25 | [MRGPRD](http://www.genenames.org/cgi-bin/gene_symbol_report?hgnc_id=29626) |  |  | C/T |  | p.Cys124Tyr | c.371G>A |
| [chr13:100634715](https://ionreporter.thermofisher.com/ir/secure/analyses/visualization.html?) | G | SNV | 0.25 | [ZIC2](http://www.genenames.org/cgi-bin/gene_symbol_report?hgnc_id=12873) |  |  | G/C |  | p.Gly133Arg | c.397G>C |
| [chr14:23871789](https://ionreporter.thermofisher.com/ir/secure/analyses/visualization.html?) | A | SNV | 0.25 | [MYH6](http://www.genenames.org/cgi-bin/gene_symbol_report?hgnc_id=7576) |  |  | A/C |  | p.Phe342Cys | c.1025T>G |
| [chr14:91792372](https://ionreporter.thermofisher.com/ir/secure/analyses/visualization.html?) | A | SNV | 0.25 | [CCDC88C](http://www.genenames.org/cgi-bin/gene_symbol_report?hgnc_id=19967) |  |  | A/T |  | p.Ile360Asn | c.1079T>A |
| [chr14:105409960](https://ionreporter.thermofisher.com/ir/secure/analyses/visualization.html?) | G | SNV | 0.25 | [AHNAK2](http://www.genenames.org/cgi-bin/gene_symbol_report?hgnc_id=20125) |  |  | G/T |  | p.Ala3943Asp | c.11828C>A |
| [chr14:105850702](https://ionreporter.thermofisher.com/ir/secure/analyses/visualization.html?) | T | SNV | 0.25 | [PACS2](http://www.genenames.org/cgi-bin/gene_symbol_report?hgnc_id=23794) |  |  | T/G |  | p.Val598Gly | c.1793T>G |
| [chr15:43827666](https://ionreporter.thermofisher.com/ir/secure/analyses/visualization.html?) | C | SNV | 0.25 | [PPIP5K1](http://www.genenames.org/cgi-bin/gene_symbol_report?hgnc_id=29023) |  |  | C/G |  | p.Gly1170Arg | c.3508G>C |
| [chr15:45353415](https://ionreporter.thermofisher.com/ir/secure/analyses/visualization.html?) | T | SNV | 0.25 | [SORD](http://www.genenames.org/cgi-bin/gene_symbol_report?hgnc_id=11184) |  |  | T/C |  | p.Phe139Ser | c.416T>C |
| [chr15:91549661](https://ionreporter.thermofisher.com/ir/secure/analyses/visualization.html?) | C | SNV | 0.25 | [VPS33B](http://www.genenames.org/cgi-bin/gene_symbol_report?hgnc_id=12712) |  |  | C/G |  | p.Gly265Arg | c.793G>C |
| [chr17:41003481](https://ionreporter.thermofisher.com/ir/secure/analyses/visualization.html?) | T | SNV | 0.25 | [AOC2 ...(2)](javascript:void(0)) |  |  | T/G |  | p.?, p.Cys41Gly | c.*1116T>G, c.121T>G |
| [chr17:73897954](https://ionreporter.thermofisher.com/ir/secure/analyses/visualization.html?) | C | SNV | 0.25 | [MRPL38](http://www.genenames.org/cgi-bin/gene_symbol_report?hgnc_id=14033) |  |  | C/G |  | p.Gly144Arg | c.430G>C |
| [chr19:19645897](https://ionreporter.thermofisher.com/ir/secure/analyses/visualization.html?) | G | SNV | 0.25 | [YJEFN3](http://www.genenames.org/cgi-bin/gene_symbol_report?hgnc_id=24785) |  |  | G/C |  | p.Gly125Arg | c.373G>C |
| [chr19:51451900](https://ionreporter.thermofisher.com/ir/secure/analyses/visualization.html?) | C | SNV | 0.25 | [KLK5](http://www.genenames.org/cgi-bin/gene_symbol_report?hgnc_id=6366) |  |  | C/A |  | p.Cys241Phe | c.722G>T |

**Additional file table S4:** **SIFT score of total SNP sorted by SNP detection filter chain**

| **Name of gene** | **Sample name** | | | |
| --- | --- | --- | --- | --- |
|  | **GBNGS008** | **GBNGS011** | **GBNGS002** | **GBNGS001** |
| *KDM3B* |  | 0 |  |  |
| *PCDH19* |  | 0 |  |  |
| *TMC7* |  | 0 |  |  |
| *FAM168B* |  | 0 |  |  |
| *PI4KB* |  | 0 |  |  |
| *TEX15* |  | 0 |  |  |
| *PLCZ1* |  | 0 |  |  |
| *QPRT* |  | 0 |  |  |
| *PVR* |  | 0 |  |  |
| *MYH6* |  | 0 |  |  |
| *RPS6KB1* |  | 0 |  |  |
| *DIDO1* |  | 0 |  |  |
| *ATP6V0C* |  | 0 |  |  |
| *FGD6* |  | 0 |  |  |
| *CEP250* |  | 0 |  |  |
| *ZNF800* |  | 0 |  |  |
| *CD101* |  | 0 |  |  |
| *HHLA1* |  | 0 |  |  |
| *OR10H4* |  | 0 |  |  |
| *SRCAP* |  | 0 |  |  |
| *POLA1* |  | 0 |  |  |
| *STAB2* |  | 0 |  |  |
| *SF3B2* |  | 0 |  |  |
| *LGI4* |  | 0 |  |  |
| *KIAA0430* |  | 0 |  |  |
| *MRPS12* |  | 0 |  |  |
| *SLC5A9* |  | 0 |  |  |
| *FAM192A* |  | 0 |  |  |
| *DRG2* |  | 0 |  |  |
| *ASAH1* |  | 0 |  |  |
| *FYN* |  | 0 |  |  |
| *DNAJC13* |  | 0 |  |  |
| *SYNGAP1* |  | 0 |  |  |
| *PADI3* |  | 0 |  |  |
| *DDX23* |  | 0 |  |  |
| *ABI2* |  | 0 |  |  |
| *SCIN* |  | 0 |  |  |
| *CDRT15* |  | 0 |  |  |
| *RAET1G* |  | 0 |  |  |
| *WWP1* |  | 0 |  |  |
| *NUDT18* |  | 0 |  |  |
| *NAALADL1* |  | 0 |  |  |
| *DNAH11* |  | 0 |  |  |
| *MAP3K3* |  | 0 |  |  |
| *RORC* |  | 0 |  |  |
| *PCLO* |  | 0 |  |  |
| *MYRF* |  | 0 |  |  |
| *AUP1* |  | 0 |  |  |
| *TRIM24* |  | 0 |  |  |
| *CTSE* |  | 0 |  |  |
| *RGSL1* |  | 0 |  |  |
| *USP39* |  | 0 |  |  |
| *PRR14L* |  | 0 |  |  |
| *RGS12* |  | 0 |  |  |
| *USP34* |  | 0 |  |  |
| *RSPO1* |  | 0 |  |  |
| *ADCY7* |  | 0 |  |  |
| *TRANK1* |  | 0 |  |  |
| *KIF24* |  | 0 |  |  |
| *KPNA7* |  | 0 |  |  |
| *TACC2* |  | 0 |  |  |
| *GBA* |  | 0 |  |  |
| *DOCK6* |  | 0 |  |  |
| *EPB41* |  | 0 |  |  |
| *C2orf81* |  | 0 |  |  |
| *ZNF853* |  | 0 |  |  |
| *NOP14* |  | 0 |  |  |
| *KANSL3* |  | 0 |  |  |
| *ABCF2* |  | 0 |  |  |
| *ABCF1* |  | 0 |  |  |
| *PDE4DIP* |  | 0 |  |  |
| *TEKT1* |  | 0 |  |  |
| *C4BPB* |  | 0 |  |  |
| *USP46* |  | 0 |  |  |
| *PPARGC1A* |  | 0 |  |  |
| *ANXA10* |  | 0 |  |  |
| *MAP1B* |  | 0 |  |  |
| *PARP6* |  | 0 |  |  |
| *NAA16* |  | 0 |  |  |
| *ALDH4A1* |  | 0 |  |  |
| *MYOM3* |  | 0 |  |  |
| *NAGPA* |  | 0 |  |  |
| *RPS2* |  | 0 |  |  |
| *MEOX1* |  | 0 |  |  |
| *RPS8* |  | 0 |  |  |
| *C18orf21* |  | 0 |  |  |
| *ADIPOR1* |  | 0 |  |  |
| *OR13D1* |  | 0 |  |  |
| *TMEM206* |  | 0 |  |  |
| *VCPIP1* |  | 0 |  |  |
| *BNIP3L* |  | 0 |  |  |
| *NDUFA9* |  | 0 |  |  |
| *SORD* |  | 0 |  |  |
| *LGALS4* |  | 0 |  |  |
| *LRP2* |  | 0 |  |  |
| *PLXNA4* |  | 0 |  |  |
| *PLG* |  | 0 |  |  |
| *COL21A1* |  | 0 |  |  |
| *PDE4B* |  | 0 |  |  |
| *PDE4C* |  | 0 |  |  |
| *ASAP1* |  | 0 |  |  |
| *CCDC85A* |  | 0 |  |  |
| *ECE2* |  | 0 |  |  |
| *MAP4K1* |  | 0 |  |  |
| *CEMIP* |  | 0 |  |  |
| *KIAA0408* |  | 0 |  |  |
| *SMCR8* |  | 0 |  |  |
| *USP9Y* |  | 0 |  |  |
| *BCL9L* |  | 0 |  |  |
| *ADCYAP1R1* |  | 0 |  |  |
| *TFAP2E* |  | 0 |  |  |
| *CPT1C* |  | 0 |  |  |
| *PEAK1* |  | 0 |  |  |
| *MRGPRD* |  | 0 |  |  |
| *VWF* |  | 0 |  |  |
| *ARID3B* |  | 0 |  |  |
| *PCDHA5* |  | 0 |  |  |
| *ABCC6* |  | 0 |  |  |
| *PTGS2* |  | 0 |  |  |
| *AOC3* |  | 0 |  |  |
| *FAM198B* |  | 0 |  |  |
| *LRPPRC* |  | 0 |  |  |
| *PLK3* |  | 0 |  |  |
| *KHNYN* |  | 0 |  |  |
| *ADAD1* |  | 0 |  |  |
| *SPINT1* |  | 0 |  |  |
| *RSBN1* |  | 0 |  |  |
| *KLHL2* |  | 0 |  |  |
| *OR2T4* |  | 0 |  |  |
| *ZNF440* |  | 0 |  |  |
| *ACSL1* |  | 0 |  |  |
| *ACSL6* |  | 0 |  |  |
| *SUPT4H1* |  | 0 |  |  |
| *PPIP5K1* |  | 0 |  |  |
| *CORO7-PAM16* |  | 0 |  |  |
| *GLIS2* |  | 0 |  |  |
| *H6PD* |  | 0 |  |  |
| *ST18* |  | 0 |  |  |
| *CHD3* |  | 0 |  |  |
| *PREX2* |  | 0 |  |  |
| *LANCL3* |  | 0 |  |  |
| *DCHS1* |  | 0 |  |  |
| *RNF40* |  | 0 |  |  |
| *TMEM44* |  | 0 |  |  |
| *SART3* |  | 0 |  |  |
| *KIAA1109* |  | 0 |  |  |
| *PEX5* |  | 0 |  |  |
| *TLK2* |  | 0 |  |  |
| *RBM22* |  | 0 |  |  |
| *PDZD2* |  | 0 |  |  |
| *ACAN* |  | 0 |  |  |
| *NES* |  | 0 |  |  |
| *GNAI1* |  | 0 |  |  |
| *FNIP1* |  | 0 |  |  |
| *SSPO* |  | 0 |  |  |
| *RRH* |  | 0 |  |  |
| *TBC1D4* |  | 0 |  |  |
| *SLC7A6* |  | 0 |  |  |
| *ASH1L* |  | 0 |  |  |
| *TSKS* |  | 0 |  |  |
| *SYNE1* |  | 0 |  |  |
| *FHOD1* |  | 0 |  |  |
| *DICER1* |  | 0 |  |  |
| *SOS1* |  | 0 |  |  |
| *WDR93* |  | 0 |  |  |
| *PKD1* |  | 0 |  |  |
| *STOML3* |  | 0 |  |  |
| *ARHGEF28* |  | 0 |  |  |
| *SREBF1* |  | 0 |  |  |
| *HIVEP3* |  | 0 |  |  |
| *KCTD2* |  | 0 |  |  |
| *OR1F1* |  | 0 |  |  |
| *TRPA1* |  | 0 |  |  |
| *CORO7* |  | 0 |  |  |
| *SERPINA4* |  | 0 |  |  |
| *CAND1* |  | 0 |  |  |
| *BLZF1* |  | 0 |  |  |
| *SVEP1* |  | 0 |  |  |
| *BRPF3* |  | 0 |  |  |
| *MSX1* |  | 0 |  |  |
| *DPY19L3* |  | 0 |  |  |
| *ADAMTSL3* |  | 0 |  |  |
| *CUBN* |  | 0 |  |  |
| *ACAP3* |  | 0 |  |  |
| *TVP23A* |  | 0 |  |  |
| *DENND1C* |  | 0 |  |  |
| *SSBP4* |  | 0 |  |  |
| *DAZAP1* |  | 0 |  |  |
| *ACTN4* |  | 0 |  |  |
| *TARBP1* |  | 0 |  |  |
| *GPR83* | 0.03 |  |  |  |
| *IL16* | 0.03 |  |  |  |
| *LILRB2* | 0.02 |  |  |  |
| *ADGRF2* | 0.01 |  |  |  |
| *MAGED2* |  | 0.05 |  |  |
| *TMEM59L* |  | 0.05 |  |  |
| *AHNAK2* |  | 0.05 |  |  |
| *PPCS* |  | 0.05 |  |  |
| *C16orf96* |  | 0.11 |  |  |
| *FAM47E* |  | 0.06 |  |  |
| *WDFY3* |  | 0.05 |  |  |
| *SASH1* |  | 0.05 |  |  |
| *ZFHX4* |  | 0.05 |  |  |
| *TGFB1* |  | 0.05 |  |  |
| *CACNA1B* |  | 0.05 |  |  |
| *MPRIP* |  | 0.05 |  |  |
| *SLC45A4* |  | 0.04 |  |  |
| *HEATR5B* |  | 0.04 |  |  |
| *CDK13* |  | 0.04 |  |  |
| *LPA* |  | 0.04 |  |  |
| *SNX27* |  | 0.04 |  |  |
| *PI4KA* |  | 0.04 |  |  |
| *KCNV1* |  | 0.04 |  |  |
| *C6orf58* |  | 0.04 |  |  |
| *CMYA5* |  | 0.04 |  |  |
| *VPS39* |  | 0.03 |  |  |
| *EXOC3L1* |  | 0.03 |  |  |
| *VPS33B* |  | 0.03 |  |  |
| *FAM47E-STBD1* |  | 0.03 |  |  |
| *GLTSCR2* |  | 0.03 |  |  |
| *FCGBP* |  | 0.03 |  |  |
| *PACS2* |  | 0.03 |  |  |
| *FAM131A* |  | 0.02 |  |  |
| *SHISA7* |  | 0.02 |  |  |
| *FRY* |  | 0.02 |  |  |
| *LENG8* |  | 0.02 |  |  |
| *ERBB3* |  | 0.02 |  |  |
| *PLIN2* |  | 0.02 |  |  |
| *B4GALT3* |  | 0.02 |  |  |
| *CTLA4* |  | 0.02 |  |  |
| *UPF1* |  | 0.02 |  |  |
| *CCDC88C* |  | 0.02 |  |  |
| *FHOD3* |  | 0.02 |  |  |
| *GRIPAP1* |  | 0.02 |  |  |
| *TNXB* | 0 | 0.01 | 0 |  |
| *OXCT1* | 0.01 | 0.01 |  |  |
| *BUB1B* |  | 0.01 |  |  |
| *MUC12* |  | 0.01 |  |  |
| *TECTA* |  | 0.01 |  |  |
| *DCTN1* |  | 0.01 |  |  |
| *IRX5* |  | 0.01 |  |  |
| *NOL6* |  | 0.01 |  |  |
| *AP5B1* |  | 0.01 |  |  |
| *SBSPON* |  | 0.01 |  |  |
| *PLEKHG4B* |  | 0.01 |  |  |
| *TTLL2* |  | 0.01 |  |  |
| *GLRA1* |  | 0.01 |  |  |
| *PLXDC1* |  | 0.01 |  |  |
| *TMC3* |  | 0.01 |  |  |
| *FBXW9* |  | 0.01 |  |  |
| *LYSMD4* |  | 0.01 |  |  |
| *IGF2BP1* |  | 0.01 |  |  |
| *HELZ* |  | 0.01 |  |  |
| *IL1F10* |  | 0.01 |  |  |
| *MATN2* |  | 0.01 |  |  |
| *DYNC1H1* |  | 0.01 |  |  |
| *ZNF577* |  | 0.01 |  |  |
| *KIAA1551* |  | 0.01 |  |  |
| *MORN3* |  | 0.01 |  |  |
| *VAT1L* |  | 0.01 |  |  |
| *EHMT2* |  | 0.01 |  |  |
| *SLFN12* |  | 0.01 |  |  |
| *MAST1* |  | 0.01 |  |  |
| *LRP1* |  | 0.01 |  |  |
| *AP3B1* |  | 0.01 |  |  |
| *C2orf78* |  | 0.01 |  |  |
| *HPN* |  | 0.01 |  |  |
| *ITPR1* |  | 0.01 |  |  |
| *RCC1* |  | 0.01 |  |  |
| *SEMA3A* |  | 0.01 |  |  |
| *SNAPC3* | 0 | 0 | 0 | 0 |
| *MMEL1* |  |  | 0 |  |
| *STAB1* |  | 0 | 0 |  |
| *NAV1* |  |  | 0 |  |
| *TNFRSF10B* |  |  | 0.03 |  |
| *RPTN* |  |  | 0 |  |
| *ARHGAP4* | 0 |  | 0 | 0 |
| *CEP68* | 0 | 0 |  | 0 |
| *UBE2QL1* |  |  |  | 0 |
| *PNKD* |  |  |  | 0 |
| *ZKSCAN5* |  |  |  | 0 |
| *CLSTN1* |  |  |  | 0 |
| *SCFD1* | 0 |  |  |  |
| *PHF14* | 0 |  |  |  |
| *DOCK8* | 0 |  |  |  |
| *BAG5* | 0 |  |  |  |
| *POLR3D* | 0 |  |  |  |
| *KIAA0922* | 0 |  |  |  |
| *ZNF415* | 0 |  |  |  |
| *AASDH* | 0 |  |  |  |
| *RHNO1* | 0 |  |  |  |
| *PPEF1* | 0 |  |  |  |
| *UBR4* | 0 |  |  |  |
| *CILP2* | 0 |  |  |  |
| *UTP20* | 0 |  |  |  |
| *HIF3A* |  | 0 |  |  |
| *ZDHHC15* |  | 0 |  |  |
| *CPE* |  | 0 |  |  |
| *ANP32E* |  | 0 |  |  |
| *AGT* |  | 0 |  |  |
| *OTUD7A* |  | 0 |  |  |
| *SMG9* |  | 0 |  |  |
| *ALMS1* |  | 0 |  |  |
| *ITPKB* |  | 0 |  |  |
| *ESPL1* |  | 0 |  |  |
| *METTL12* |  | 0 |  |  |
| *KDM2B* |  | 0 |  |  |
| *SLC35E2B* |  | 0 |  |  |
| *OPN1SW* |  | 0 |  |  |
| *SLC38A2* |  | 0 |  |  |
| *TAS2R14* |  | 0 |  |  |
| *SLC45A3* |  | 0 |  |  |
| *MUC16* |  | 0 |  |  |
| *COL7A1* |  | 0 |  |  |
| *GADD45GIP1* |  | 0 |  |  |
| *STARD9* |  | 0 |  |  |
| *GATB* |  | 0 |  |  |
| *NTHL1* |  | 0 |  |  |
| *EGFLAM* |  | 0 |  |  |
| *ANO4* |  | 0 |  |  |
| *EDC3* |  | 0 |  |  |
| *DENND4B* |  | 0 |  |  |
| *TMEM132D* |  | 0 |  |  |
| *XPO6* |  | 0 |  |  |
| *ZIC2* |  | 0 |  |  |
| *JAK3* |  | 0 |  |  |
| *COL4A3* |  | 0 |  |  |
| *BLK* |  | 0 |  |  |
| *BRF1* |  | 0 |  |  |
| *BNIP2* |  | 0 |  |  |
| *RPS6KA1* |  | 0 |  |  |
| *KCNH2* |  | 0 |  |  |
| *GIT2* |  | 0 |  |  |
| *SLC22A3* |  | 0 |  |  |
| *NOV* |  | 0 |  |  |
| *GIGYF1* |  | 0 |  |  |
| *PELP1* |  | 0 |  |  |
| *HMGXB3* |  | 0 |  |  |
| *ENPEP* |  | 0 |  |  |
| *ASPG* |  | 0 |  |  |
| *PLRG1* |  | 0 |  |  |
| *KNTC1* |  | 0 |  |  |
| *ADGRB1* |  | 0 |  |  |
| *KIF21B* |  | 0 |  |  |
| *SZT2* |  | 0 |  |  |
| *IL17F* |  | 0 |  |  |
| *LAMA3* |  | 0 |  |  |
| *DGAT2L6* |  | 0 |  |  |
| *TAF1C* |  | 0 |  |  |
| *ALK* |  | 0 |  |  |
| *FAM20A* |  | 0 |  |  |
| *ATP1A3* |  | 0 |  |  |
| *GREB1L* |  | 0 |  |  |
| *B4GALNT1* |  | 0 |  |  |
| *PGLYRP2* |  | 0 |  |  |
| *WDR81* |  | 0 |  |  |
| *SPTBN4* |  | 0 |  |  |
| *KAZN* |  | 0 |  |  |
| *LTBP1* |  | 0 |  |  |
| *FBN3* |  | 0 |  |  |
| *ST6GALNAC1* |  | 0 |  |  |
| *THAP9* |  | 0 |  |  |
| *MAPKBP1* |  | 0 |  |  |
| *ANK2* |  | 0 |  |  |
| *ZNF541* |  | 0 |  |  |
| *ZNF462* |  | 0 |  |  |
| *UFC1* |  | 0 |  |  |
| *REXO1* |  | 0 |  |  |
| *3-Mar* |  | 0 |  |  |
| *ADAMTS2* |  | 0 |  |  |
| *KRT72* |  | 0 |  |  |
| *FNDC1* |  | 0 |  |  |
| *RELN* |  | 0 |  |  |
| *IL36B* |  | 0 |  |  |
| *JAKMIP2* |  | 0 |  |  |
| *MRPL38* |  | 0 |  |  |
| *RPH3A* |  | 0 |  |  |
| *KLK5* |  | 0 |  |  |
| *KIRREL2* |  | 0 |  |  |
| *CENPV* |  | 0 |  |  |
| *ALDH5A1* |  | 0 |  |  |
| *ALG3* |  | 0 |  |  |
| *CEP104* |  | 0 |  |  |
| *VWC2L* |  | 0 |  |  |
| *NCKAP5L* |  | 0 |  |  |
| *LHCGR* |  | 0 |  |  |
| *YJEFN3* |  | 0 |  |  |
| *CCDC121* |  | 0 |  |  |
| *PARPBP* |  | 0 |  |  |
| *TULP3* |  | 0 |  |  |
| *CCDC129* |  | 0 |  |  |
| *CDC42BPG* |  | 0 |  |  |
| *GCC1* |  | 0 |  |  |
| *TNS3* |  | 0 |  |  |
| *ASTN1* |  | 0 |  |  |
| *OR8D2* |  | 0 |  |  |
| *SCMH1* |  | 0 |  |  |
| *IRF3* |  | 0 |  |  |
| *PCNX1* |  | 0 |  |  |
| *SH3TC1* |  | 0 |  |  |

**Additional file table S5:** **SIFT score of total MNV sorted by MNV detection filter chain**

| Gene | Sample name | | | |
| --- | --- | --- | --- | --- |
|  | GBNGS002 | GBNGS001 | GBNGS008 | GBNGS011 |
| *PSD2* |  |  |  | 0 |
| *OR2A12* |  |  |  | 0 |
| *PTCD1* |  |  |  | 0 |
| *GTF3A* |  |  |  | 0 |
| *CAMKK2* |  |  |  | 0 |
| *ANPEP* |  |  |  | 0 |
| *IL11RA* |  |  |  | 0 |
| *VWF* |  |  |  | 0 |
| *DMWD* |  |  |  | 0 |
| *SOAT2* |  |  |  | 0 |
| *ZNF333* |  |  |  | 0 |
| *EHMT2* |  |  |  | 0 |
| *ALS2CR11* |  |  |  | 0 |
| *MYH2* |  |  |  | 0 |
| *PRR14L* |  |  |  | 0 |
| *NCOA1* |  |  |  | 0 |
| *JMJD6* |  |  |  | 0 |
| *KLK12* |  |  |  | 0 |
| *SLC22A23* |  |  |  | 0 |
| *KRT83* |  |  |  | 0 |
| *KNTC1* |  |  |  | 0 |
| *GPN1* |  |  |  | 0 |
| *KRT6A* |  |  |  | 0 |
| *SLC6A12* |  |  |  | 0 |
| *ADGRB2* |  |  |  | 0 |
| *RANBP2* |  |  |  | 0 |
| *TRIO* |  |  |  | 0 |
| *DAGLB* |  |  |  | 0 |
| *ICAM1* |  |  |  | 0 |
| *MAST2* |  |  |  | 0 |
| *SH2B1* |  |  |  | 0 |
| *FADS1* |  |  |  | 0 |
| *ACHE* |  |  |  | 0 |
| *FOXB2* |  |  |  | 0 |
| *TAF1C* |  |  |  | 0 |
| *TJP3* |  |  |  | 0 |
| *CD101* |  |  |  | 0 |
| *KEL* |  |  |  | 0 |
| *TRAFD1* |  |  |  | 0 |
| *GPBP1L1* |  |  |  | 0 |
| *EDC3* |  |  |  | 0 |
| *TACC2* |  |  |  | 0 |
| *HHLA1* |  |  |  | 0 |
| *NTN4* |  |  |  | 0 |
| *STOML2* |  |  |  | 0 |
| *ANKRD12* |  |  |  | 0 |
| *WDCP* |  |  |  | 0 |
| *BABAM1* |  |  |  | 0 |
| *PARD3B* |  |  |  | 0 |
| *ZNF134* |  |  |  | 0 |
| *LRRC8E* |  |  |  | 0 |
| *RBM4* |  |  |  | 0 |
| *CHPF2* |  |  |  | 0 |
| *LOXHD1* |  |  |  | 0 |
| *CLGN* |  |  |  | 0 |
| *ZNF326* |  |  |  | 0 |
| *NOC4L* |  |  |  | 0 |
| *DSCAM* |  |  |  | 0 |
| *CLCNKB* |  |  |  | 0 |
| *DCLRE1B* |  |  |  | 0 |
| *DRG2* |  |  |  | 0 |
| *TLN2* |  |  |  | 0 |
| *UHMK1* |  |  |  | 0 |
| *CNOT1* |  |  |  | 0 |
| *HSD17B3* |  |  |  | 0 |
| *SPOCD1* |  |  |  | 0 |
| *NOS2* |  |  |  | 0 |
| *KCND2* |  |  |  | 0 |
| *LTBP3* |  |  |  | 0 |
| *IPO9* |  |  |  | 0 |
| *ADAT1* |  |  |  | 0 |
| *PRKCG* |  |  |  | 0 |
| *PLAT* |  |  |  | 0 |
| *GPC6* |  |  |  | 0 |
| *GTPBP2* |  |  |  | 0 |
| *RBM14-RBM4* |  |  |  | 0 |
| *C8orf86* |  |  |  | 0 |
| *SHANK1* |  |  |  | 0 |
| *ZNF296* |  |  |  | 0 |
| *CABIN1* |  |  |  | 0 |
| *C7orf43* |  |  |  | 0 |
| *TXNDC11* |  |  |  | 0 |
| *ZNF469* |  |  |  | 0 |
| *TOM1L2* |  |  |  | 0 |
| *ADH1A* |  |  |  | 0 |
| *MUL1* |  |  |  | 0 |
| *SMPDL3B* |  |  |  | 0.28 |
| *RHBDL3* |  |  |  | 1 |
| *MUC2* |  |  |  | 0.24 |
| *MT4* |  |  |  | 0.17 |
| *RIMBP2* |  |  |  | 0.16 |
| *C16orf96* |  |  |  | 0.11 |
| *NPR3* |  |  |  | 0.09 |
| *FRY* |  |  |  | 0.08 |
| *IRF8* |  |  |  | 0.05 |
| *H6PD* |  |  |  | 0.05 |
| *E2F6* |  |  |  | 0.05 |
| *ERGIC2* |  |  |  | 0.04 |
| *ALKBH7* |  |  |  | 0.04 |
| *SNRPA1* |  |  |  | 0.04 |
| *CDK13* |  |  |  | 0.04 |
| *SFSWAP* |  |  |  | 0.04 |
| *RP1L1* |  |  |  | 0.03 |
| *SLC12A9* |  |  |  | 0.03 |
| *PKD1* |  |  |  | 0.03 |
| *CHRD* |  |  |  | 0.03 |
| *MAGED1* |  |  |  | 0.02 |
| *DDX43* |  |  |  | 0.02 |
| *B4GALT3* |  |  |  | 0.02 |
| *TRIM2* |  |  |  | 0.02 |
| *ATR* |  |  |  | 0.02 |
| *PKD2* |  |  |  | 0.02 |
| *KEAP1* |  |  |  | 0.02 |
| *ZMYM3* |  |  |  | 0.02 |
| *ARSD* |  |  |  | 0.02 |
| *ATP5J2-PTCD1* |  |  |  | 0.01 |
| *FAM131A* |  |  |  | 0.01 |
| *USP38* |  |  |  | 0.01 |
| *IFT46* |  |  |  | 0.01 |
| *HSPG2* |  |  |  | 0.01 |
| *PHLPP2* |  |  |  | 0.01 |
| *ZNF609* |  |  |  | 0.01 |
| *JAML* |  |  |  | 0.01 |
| *CUX2* |  |  |  | 0.01 |
| *SLC22A3* |  |  |  | 0.01 |
| *CREBBP* |  |  |  | 0.01 |
| *PHF1* |  |  |  | 0.01 |
| *EPHA2* |  |  |  | 0.01 |
| *RRH* |  |  |  | 0.01 |
| *AP3D1* |  |  |  | 0.01 |
| *ATP6V0B* |  |  |  | 0.01 |
| *CEP250* |  |  |  | 0.01 |
| *GRIPAP1* |  |  |  | 0.01 |
| *NOL6* |  |  |  | 0.01 |
| *VWA3B* |  |  |  | 0.01 |
| *CUBN* |  |  |  | 0.01 |
| *AHNAK2* |  |  |  | 0.01 |
| *KLHL5* |  |  | 0.01 |  |
| *MEGF6* |  |  | 0.01 |  |
| *AIM1L* | 0 | 0 |  |  |
| *ACOT4* | 0 |  |  |  |
| *DDX60* | 0 |  |  |  |
| *NLRX1* | 0 |  |  |  |
| *FAM189B* | 0.01 |  |  |  |
| *ERICH6B* |  | 0 |  |  |
| *PIF1* |  | 0.02 |  |  |
| *SON* |  |  | 0 |  |
| *DNAH10* |  |  |  | 0 |
| *DNAH11* |  |  |  | 0 |
| *TGDS* |  |  |  | 0 |
| *LYPLA1* |  |  |  | 0 |
| *SCG5* |  |  |  | 0 |
| *GLG1* |  |  |  | 0 |
| *RAPGEF2* |  |  |  | 0 |
| *SLC30A1* |  |  |  | 0 |
| *TRIM62* |  |  |  | 0 |
| *ADAMTS7* |  |  |  | 0 |
| *GABRB3* |  |  |  | 0 |
| *CNGB1* |  |  |  | 0 |
| *MAP3K6* |  |  |  | 0 |
| *CACNA1F* |  |  |  | 0 |
| *SLC34A1* |  |  |  | 0 |
| *NRCAM* |  |  |  | 0 |
| *ESPL1* |  |  |  | 0 |
| *ZNF786* |  |  |  | 0 |
| *PFAS* |  |  |  | 0 |
| *DYSF* |  |  |  | 0 |
| *WNK2* |  |  |  | 0 |
| *EXTL3* |  |  |  | 0 |
| *MUC6* |  |  |  | 0 |
| *CCDC78* |  |  |  | 0 |
| *FCGBP* |  |  |  | 0 |
| *SERPINB8* |  |  |  | 0 |
| *EYS* |  |  |  | 0 |
| *MEPCE* |  |  |  | 0 |
| *KCNN3* |  |  |  | 0 |
| *MUC16* |  |  |  | 0 |
| *SLC25A29* |  |  |  | 0 |
| *TNS1* |  |  |  | 0 |
| *KPTN* |  |  |  | 0 |
| *ADCY7* |  |  |  | 0 |
| *TRANK1* |  |  |  | 0 |
| *TCAP* |  |  |  | 0 |
| *ZBTB22* |  |  |  | 0 |
| *MYO15A* |  |  |  | 0 |
| *PLAGL1* |  |  |  | 0 |
| *NUP205* |  |  |  | 0 |
| *RASGRP2* |  |  |  | 0 |
| *MRPL24* |  |  |  | 0 |
| *LRFN5* |  |  |  | 0 |
| *C16orf72* |  |  |  | 0 |
| *TP53BP1* |  |  |  | 0 |
| *MGST3* |  |  |  | 0 |
| *CEP68* |  |  |  | 0 |
| *DENND4B* |  |  |  | 0 |
| *MAT2A* |  |  |  | 0 |
| *ARID4B* |  |  |  | 0 |
| *SMURF1* |  |  |  | 0 |
| *FAM161B* |  |  |  | 0 |
| *ZNF606* |  |  |  | 0 |
| *LILRB2* |  |  |  | 0 |
| *ABCF1* |  |  |  | 0 |
| *SNRNP25* |  |  |  | 0 |
| *ADAR* |  |  |  | 0 |
| *FBXL14* |  |  |  | 0 |
| *SF3A3* |  |  |  | 0 |
| *PDLIM4* |  |  |  | 0 |
| *TRIOBP* |  |  |  | 0 |
| *STAG3* |  |  |  | 0 |
| *PARP6* |  |  |  | 0 |
| *ANKLE2* |  |  |  | 0 |
| *HERC1* |  |  |  | 0 |
| *HERC2* |  |  |  | 0 |
| *MIA3* |  |  |  | 0 |
| *SEZ6L2* |  |  |  | 0 |
| *KIAA1109* |  |  |  | 0 |
| *DNAH2* |  |  |  | 0 |
| *LCT* |  |  |  | 0 |
| *ALDH4A1* |  |  |  | 0 |

**Additional file table S6: Splice variant impact of 42 genes incurred with frameshift deletion mutation due to MNV**

| Gene | **Sample name** | | | |
| --- | --- | --- | --- | --- |
|  | **GBNGS002** | **GBNGS001** | **GBNGS011** | **GBNGS008** |
| *ATR* |  |  | 7 |  |
| *DNAH10* |  |  | 7 |  |
| *ARID4B* |  |  | 7 |  |
| *ADAMTS7* |  |  | 7 |  |
| *JMJD6* |  |  | 7 |  |
| *MAP3K6* |  |  | 7 |  |
| *KLK12* |  |  | 7 |  |
| *SF3A3* |  |  | 7 |  |
| *B4GALT3* |  |  | 7 |  |
| *HSD17B3* |  |  | 7 |  |
| *SCG5* |  |  | 7 |  |
| *PFAS* |  |  | 7 |  |
| *ARSD* |  |  | 7 |  |
| *NOS2* |  |  | 7 |  |
| *KCND2* |  |  | 7 |  |
| *CUBN* |  |  | 7 |  |
| *MUC2* |  |  | 7 |  |
| *WDCP* |  |  | 7 |  |
| *AHNAK2* |  |  | 7 |  |
| *SLC25A29* |  |  | 7 |  |
| *DNAH2* |  |  | 7 |  |
| *TJP3* |  |  | 7 |  |
| *MEPCE* |  |  | 7 |  |
| *PKD2* |  |  | 7 |  |
| *TXNDC11* |  |  | 7 |  |
| *GTF3A* |  |  | 7 |  |
| *MYO15A* |  |  | 7 |  |
| *PHF1* |  |  | 7 |  |
| *RBM4* |  |  | 7 |  |
| *RBM14-RBM4* |  |  | 7 |  |
| *ATP5J2-PTCD1* |  |  | 7 |  |
| *PTCD1* |  |  | 7 |  |
| *IFT46* |  |  | 7 |  |
| *NRCAM* |  |  | 7 |  |
| *CHPF2* |  |  | 7 |  |
| *SH2B1* |  |  | 7 |  |
| *DDX60* | 7 |  |  |  |
| *PIF1* |  | 7 |  |  |
| *METTL23* |  |  | 0 |  |
| *SNN* |  |  | 0 |  |
| *MTIF3* |  |  | 0 |  |
| *KLHL5* |  |  |  | 7 |

**Additional file table S7: SIFT score of total INDEL sorted by INDEL detection filter chain**

| Gene | Sample name | | | |
| --- | --- | --- | --- | --- |
|  | GBNGS002 | GBNGS001 | GBNGS011 | GBNGS008 |
| *ADAMTS7* |  |  | 0 |  |
| *ADAMTSL3* |  |  | 0 |  |
| *AHNAK2* |  |  | 0.01 |  |
| *ARID4B* |  |  | 0 |  |
| *ARSD* |  |  | 0.02 |  |
| *ATP5J2-PTCD1* |  |  | 0.26 |  |
| *ATR* |  |  | 0.02 |  |
| *B4GALT3* |  |  | 0.12 |  |
| *BAG5* |  |  |  | 0 |
| *C2orf42* |  |  | 0 |  |
| *CCDC78* |  |  | 0 |  |
| *CEMIP* |  |  | 0 |  |
| *CENPV* |  |  | 0 |  |
| *CFAP69* |  |  | 0.01 |  |
| *CHPF2* |  |  | 0 |  |
| *CILP2* |  |  |  | 0 |
| *COL21A1* |  |  | 0 |  |
| *CUBN* |  |  | 0.01 |  |
| *DDX60* | 0 |  |  |  |
| *DNAH10* |  |  | 0 |  |
| *DNAH2* |  |  | 0 |  |
| *DYNC1H1* |  |  | 0.01 |  |
| *EHMT2* |  |  | 0 |  |
| *EPB41* |  |  | 0 |  |
| *EXOSC10* |  |  | 0 |  |
| *EXTL3* |  |  | 0 |  |
| *EYS* |  |  | 0 |  |
| *FAM65B* |  |  | 0 |  |
| *FBN3* |  |  | 0 |  |
| *FBXL14* |  |  | 0 |  |
| *FOXB2* |  |  | 0 |  |
| *GTF3A* |  |  | 0 |  |
| *HSD17B3* |  |  | 0 |  |
| *IFT46* |  |  | 0.01 |  |
| *IRF8* |  |  | 0.05 |  |
| *JMJD6* |  |  | 0 |  |
| *KCND2* |  |  | 0 |  |
| *KCNV1* |  |  | 0.04 |  |
| *KLHL5* |  |  |  | 0.01 |
| *KLK12* |  |  | 0 |  |
| *LANCL3* |  |  | 0 |  |
| *LEXM* |  |  | 0 |  |
| *LTBP1* |  |  | 0 |  |
| *MAP1S* |  |  | 0 |  |
| *MAP3K6* |  |  | 0 |  |
| *MEGF6* |  |  |  | 0.01 |
| *MEPCE* |  |  | 0 |  |
| *MORN1* |  |  | 0 |  |
| *MT4* |  |  | 0.17 |  |
| *MUC2* |  |  | 0.24 |  |
| *MYO15A* |  |  | 0.03 |  |
| *NOC4L* |  |  | 0 |  |
| *NOS2* |  |  | 0 |  |
| *NRCAM* |  |  | 0 |  |
| *NUDT18* |  |  | 0 |  |
| *OR10H4* |  |  | 0 |  |
| *PDX1* |  |  | 0 |  |
| *PFAS* |  |  | 0 |  |
| *PHF1* |  |  | 0.01 |  |
| *PIF1* |  | 0.02 |  |  |
| *PKD2* |  |  | 0.02 |  |
| *PPCS* |  |  | 0.05 |  |
| *PRR14L* |  |  | 0 |  |
| *PRR25* |  |  | 0 |  |
| *PTCD1* |  |  | 0.41 |  |
| *RBM14-RBM4* |  |  | 0 |  |
| *RBM4* |  |  | 0 |  |
| *RCC1* |  |  | 0.01 |  |
| *RELN* |  |  | 0 |  |
| *SCG5* |  |  | 0 |  |
| *SF3A3* |  |  | 0 |  |
| *SH2B1* |  |  | 0 |  |
| *SH3TC1* |  |  | 0 |  |
| *SHE* |  |  | 0.04 |  |
| *SLC25A29* |  |  | 0 |  |
| *SLC30A1* |  |  | 0 |  |
| *SLC35E2B* |  |  | 0 |  |
| *SNAPC3* |  |  |  | 0 |
| *SRCAP* |  |  | 0 |  |
| *ST6GALNAC2* |  |  | 0.05 |  |
| *SZT2* |  |  | 0 |  |
| *TECTA* |  |  | 0.01 |  |
| *TJP3* |  |  | 0 |  |
| *TRIOBP* |  |  | 0 |  |
| *TVP23A* |  |  | 0 |  |
| *TVP23B* |  |  | 0 |  |
| *TXNDC11* |  |  | 0 |  |
| *USP46* |  |  | 0 |  |
| *WDCP* |  |  | 0 |  |
| *XPO6* |  |  | 0 |  |
| *ZNF333* |  |  | 0 |  |
| *USP46* |  |  | 7 |  |
| *WDCP* |  |  | 7 |  |
| *XPO6* |  |  | 7 |  |
| *ZNF333* |  |  | 5 |  |
